# Responsiveness of the domain climbing up and going down stairs of the Functional Evaluation scale for Duchenne Muscular Dystrophy: a one-year follow-up

**DOI:** 10.1590/bjpt-rbf.2014.0178

**Published:** 2016-06-16

**Authors:** Priscila S. Albuquerque, Mariana C. Voos, Mariene S. Simões, Joyce Martini, Carlos B. M. Monteiro, Fatima A. Caromano

**Affiliations:** 1 Programa de Pós-graduação em Ciências da Reabilitação, Laboratório de Fisioterapia e Comportamento, Faculdade de Medicina, Universidade de São Paulo (USP), São Paulo, SP, Brazil

**Keywords:** Duchenne muscular dystrophy, disability evaluation, physical therapy modalities, motor skills

## Abstract

**Objective:**

To determine the responsiveness of the domain climbing up and going down stairs of the Functional Evaluation Scale for Duchenne Muscular Dystrophy (FES-DMD-D3) in a one-year follow-up study.

**Method:**

The study included 26 patients with DMD. Effect Size (ES) and Standardized Response Mean (SRM) described the scale’s responsiveness.

**Results:**

For climbing up stairs, ES showed that responsiveness was low in the three-month assessments (0.26; 0.35; 0.13; 0.17), low to moderate in the six-month assessments (0.58; 0.48; 0.33), moderate in the nine-month assessments (0.70; 0.68), and high in the 12-month assessment (0.88). SRM showed that responsiveness was low in the three-month assessments (0.29; 0.38; 0.18; and 0.19), low to moderate in the six-month assessments (0.59; 0.51; 0.36), moderate in the nine-month assessments (0.74 and 0.70), and high in the 12-month assessment (0.89). For going down stairs, ES showed that responsiveness was low in the three- and six-month assessments (0.16; 0.25; 0.09; 0.08 and 0.48; 0.35; 0.18, respectively), low to moderate in the nine-month assessments (0.59; 0.44), and moderate in the 12-month assessment (0.71). SRM showed that responsiveness was low in the three- and six-month assessments (0.25; 0.35; 0.12; 0.09 and 0.47; 0.38; 0.21, respectively), low to moderate in the nine-month assessment (0.62; 0.49), and moderate in the 12-month assessment (0.74).

**Conclusion:**

Climbing up stairs should be assessed at intervals of nine months or longer, when responsiveness is moderate to high. Going down stairs should be assessed annually because moderate responsiveness was observed in this period.

## BULLET POINTS

- The study describes the responsiveness of the domain climbing up and going down stairs of the FES-DMD-D3 in children with DMD.
- Descriptive indicators of the progression of children with DMD were determined in the 12-month follow-up assessment of climbing stairs.

## Introduction

Duchenne muscular dystrophy (DMD) is a recessive genetic disorder, caused by the alteration of the gene that encodes dystrophin protein, which is essential for maintaining the muscle cell membrane. The affected gene is located on the short arm of chromosome X, locusXp21^1^ ^-^ ^3^. DMD is characterized by generalized and irreversible progressive deterioration of muscular strength. Symptoms appear when children are three to five years old. Lower limbs are affected first, leading to difficulty climbing steps, running, and getting up from the floor, frequent falls, and pseudohypertrophy of the calf muscles. When children are 10 to 12 years old, patients lose gait and show upper limb and respiratory muscle weakness, spine deformities, and heart dysfunction^1^ ^-^ ^3^.

Evaluation scales are widely used in clinical practice and research for the functional assessment of patients with DMD, such as the Hammersmith Functional Motor Scale^4^, Motor Function Measure Scale (MFM)^5^, Egen Klassifikation (EK)^6^, North Star Ambulatory Assessment^7^, and Functional Evaluation Scale for Duchenne Muscular Dystrophy (FES-DMD)^8^ ^-^ ^11^.

The FES-DMD is the only functional evaluation scale that describes compensatory movements, which are a consequence of muscular strength loss. It consists of four domains, sitting and rising from the chair (domain 1), walking (domain 2), climbing up and going down stairs (domain 3), and sitting and standing up from the floor (domain 4). Previous studies have shown that its intra- and inter-rater reliability ranges from good to excellent^8^ ^-^ ^11^.

The evaluation of climbing up stairs is divided into five stages. Phase I or preparation in standing position consists of three subphases. Phase II or propulsion consists of eight subphases. Phase III or pelvic swing consists of seven subphases. Phase IV or lower limb swing consists of three subphases. Phase V or support consists of four subphases^10^. The evaluation of going down stairs is divided into four phases. Phase I or preparation in standing position is composed of three subphases. Phase II or propulsion is composed of five subphases. Phase III or balance comprises ten subphases. Phase IV or support consists of nine subphases^10^. This clearly shows that climbing up and going down stairs are complex activities that require muscle synergy and stability; however, research on these activities is limited to the analysis of falls during descent, skill development and learning, or timed performance. The most comprehensive study involving the kinesiological aspects of this activity was conducted in healthy adults by McFadyen and Winter and became a reference for subsequent studies^12^.

Despite excellent results regarding the reliability of the FES-DMD, appropriate reassessment intervals has not yet been established. Thus, it is necessary to measure the responsiveness of each domain of the FES-DMD. Responsiveness is an index of sensitivity that detects the clinical change over time due to disease progression or clinical intervention^13^ ^-^ ^15^. This study specifically analyzed the responsiveness of the domain climbing up and going down stairs (FES-DMD-D3) and identified the appropriate intervals between evaluations in a one-year follow-up.

## Method

### Experimental design

Observational, longitudinal, and retrospective study approved by the Research Ethics Committee of Faculdade de Medicina da Universidade de São Paulo (USP), São Paulo, SP, Brazil, process number 435/13. The subjects’ parents or legal guardians signed an informed consent form prior to participation.

### Subjects

The image bank of Laboratório de Miopatias do Instituto de Biociências da USP was used. Twenty-six children with molecular diagnosis of DMD participated in this study. They were taking steroids regularly and were classified as 1 to 3 on the Vignos Scale. Their mean age was 8.1±1.8 years old, mean height 1.39±0.17, and mean weight 40.8±10.4 kg (mean±SD). Each child was evaluated four times in a 12-month period at intervals of three, six, nine, and 12 months. Four patients who did not attend to all assessments were excluded.

### Procedures

We evaluated 23 videos of the subjects climbing up stairs and 26 videos of the subjects going down stairs. FES-DMD-DATA software was used for the assessment.

Responsiveness was assessed at three-month intervals (0 to 3, 3 to 6, 6 to 9, and 9 to 12 months), six-month intervals (0 to 6, 3 to 9, and 6 to 12 months), nine-month intervals (0 to 9 and 3 to 12), and at a 12-month interval (0 to 12 months). This methodology was based on previous responsiveness studies^16^ ^-^ ^22^.

Recommendations about the best responsiveness evaluation method are still lacking in the literature. Many approaches have been used but none is considered standard. Responsiveness is assessed using different indicators such as Effect Size (ES) and Standardized Response Mean (SRM)^16^ ^-^ ^22^. According to Mehrholz et al.^23^, the SRM can better reflect the individual changes than the ES test. Samsa et al.^24^ reported that the ES should be interpreted with caution. Other authors have recommended that both should be combined to avoid misinterpretations^23^ ^,^ ^24^.

Following these recommendations, ES and SRM were used. ES was calculated using the formula: ES= Mx - M0/ DPb. Mx was the mean follow-up assessment score in time x, M0 was the mean evaluation score at time 0 (initial evaluation) and DPb was the standard deviation of the initial evaluation. SRM was calculated by the formula: SRM = Mx - M0/ DP(Mx - M0). The denominator was the standard deviation of the difference between Mx and M0^22^.

The analysis considered Cohen's criteria, which established that coefficients >0.20 and <0.50 indicated low responsiveness, coefficients >0.5 and <0.8 indicated moderate responsiveness and coefficients >0.8 reflected high responsiveness^25^.

## Results

### Climbing up stairs

Table 1 shows the ES of each phase, considering the evaluation intervals and the total score obtained in the domain climbing up stairs of the FES-DMD-D3. Climbing up stairs showed low responsiveness in all four three-month assessments (0.26, 0.35, 0.13 and 0.17). Responsiveness was moderate in one of the six-month assessments (0.58) and low in the other two six-month assessments (0.48 and 0.33). In the nine-month assessments, responsiveness was moderate (0.70 and 0.68). In the 12-month assessment, responsiveness was high (0.88) (Table 1).

**Table 1:** Responsiveness analysis (Effect Size) of climbing up stairs.

**Evaluation**	**0 X 3**	**3 X 6**	**6 X 9**	**9X12**	**0 X 6**	**3 X 9**	**6 X 12**	**0 X 9**	**3 X 12**	**0 X 12**
**Phases**	**3-month intervals**				**6-month intervals**			**9-month intervals**		**1-year** **follow-up**
Phase 1 - preparation	0.42	0.14	0.02	0.09	0.54	0.17	0.11	0.57	0.28	0.66
Phase 2 - propulsion	0.30	0.38	0.17	0.13	0.74	0.55	0.33	0.93	0.70	1.09
Phase 3 - pelvic balance	0.20	0.14	–0.05	0.14	0.33	0.09	0.10	0.29	0.23	0.41
Phase 4 - swing	0.44	0.20	0.37	0.14	0.11	0.51	0.50	0.44	0.61	0.56
Phase 5 - support	0.04	0.32	0.08	0.16	0.34	0.41	0.24	0.43	0.61	0.61
**Total score**	0.26	0.35	0.13	0.17	0.58	0.48	0.33	0.70	0.68	0.88

Coefficients >0.20 and <0.50 indicate low responsiveness, coefficients >0.5 and <0.8 indicate moderate responsiveness, and coefficients >0.8 denote high responsiveness.

Table 2 shows the SRM of each stage, considering the evaluation intervals and the total score obtained in the FES-DMD-D3. Climbing up stairs responsiveness was classified as low in the four three-month assessments (0.29, 0.38, 0.18, and 0.19). At six-month intervals, responsiveness was moderate in two assessments (0.59 and 0.51) and low in the first assessment (0.36). In the nine-month assessments, responsiveness was moderate (0.74 and 0.70). In the 12-month assessment, responsiveness was high (0.89) (Table 2).

**Table 2:** Responsiveness analysis (Standardized Response Mean) of climbing up stairs.

**Evaluation**	**0 X 3**	**3 X 6**	**6 X 9**	**9X12**	**0 X 6**	**3 X 9**	**6 X 12**	**0 X 9**	**3 X 12**	**0 X 12**
**Phases**	**3-month intervals**				**6-month intervals**			**9-month intervals**		**1-year** **follow-up**
Phase 1 - preparation	0.44	0.16	0.03	0.11	0.56	0.17	0.13	0.58	0.28	0.67
Phase 2 - propulsion	0.33	0.39	0.19	0.15	0.75	0.56	0.34	0.96	0.72	1.10
Phase 3 - pelvic balance	0.21	0.15	0.05	0.16	0.32	0.10	0.11	0.31	0.25	0.43
Phase 4 - swing	0.46	0.23	0.39	0.14	0.13	0.53	0.50	0.44	0.62	0.56
Phase 5 - support	0.05	0.35	0.08	0.15	0.35	0.42	0.26	0.42	0.63	0.63
**Total score**	0.29	0.38	0.18	0.19	0.59	0.51	0.36	0.74	0.70	0.89

Coefficients >0.20 and <0.50 indicate low responsiveness, coefficients >0.5 and <0.8 indicate moderate responsiveness, and coefficients >0.8 denote high responsiveness.

### Climbing down stairs

Table 3 shows the ES of each stage, considering the evaluation intervals and the total score obtained in the FES-DMD-D3. For climbing down stairs, the three-month assessments (0.16, 0.25, 0.09, and 0.08) and six-month assessments (0.48, 0.35, and 0.18) showed low responsiveness. In the nine-month assessments, responsiveness was moderate in one assessment (0.59) and low in the other (0.44). In the 12-month assessment, responsiveness was moderate (0.71) (Table 3).

**Table 3:** Responsiveness analysis (Effect Size) of going down stairs.

**Evaluation**	0 X 3	3 X 6	6 X 9	9X12	0 X 6	3 X 9	6 X 12	0 X 9	3 X 12	0 X 12
**Phases**	**3-month intervals**				**6-month intervals**			**9-month intervals**		**1-year** **follow-up**
Phase 1 - preparation	0.27	0.27	0.06	0.02	0.55	0.33	0.09	0.61	0.35	0.64
Phase 2 - propulsion	0.38	0.11	0.09	0.03	0.59	0.19	0.13	0.72	0.22	0.77
Phase 3 - swing	0.10	0.39	0.11	0.08	0.36	0.53	0.19	0.50	0.64	0.60
Phase 4 - support	–0.08	0.22	0.05	0.13	0.12	0.29	0.19	0.18	0.48	0.34
**Total score**	0.16	0.25	0.09	0.08	0.48	0.35	0.18	0.59	0.44	0.71

Coefficients >0.20 and <0.50 indicate low responsiveness, coefficients >0.5 and <0.8 indicate moderate responsiveness, and coefficients >0.8 denote high responsiveness.

Table 4 shows the SRM of each phase, considering the evaluation intervals and the total score of the FES-DMD-D3. Climbing down stairs showed low responsiveness in the three-month assessments (0.25, 0.35, 0.12, and 0.09) and in the six-month assessments (0.47, 0.38, and 0.21). In the nine-month assessments, responsiveness was moderate (0.62) and low (0.49). In 12-month assessment, responsiveness was moderate (0.74) (Table 4).

**Table 4:** Responsiveness analysis (Standardized Response Mean) of going down stairs.

**Evaluation**	0 X 3	3 X 6	6 X 9	9X12	0 X 6	3 X 9	6 X 12	0 X 9	3 X 12	0 X 12
**Phases**	**3-month intervals**				**6-month intervals**			**9-month intervals**		**1 year** **follow-up**
Phase 1 - preparation	0.31	0.32	0.15	0.04	0.58	0.35	0.11	0.63	0.38	0.66
Phase 2 - propulsion	0.42	0.15	0.12	0.06	0.61	0.21	0.15	0.74	0.24	0.79
Phase 3 - swing	0.20	0.42	0.16	0.12	0.38	0.55	0.20	0.52	0.66	0.63
Phase 4 - support	0.12	0.25	0.07	0.15	0.15	0.31	0.21	0.20	0.50	0.36
**Total score**	0.25	0.35	0.12	0.09	0.47	0.38	0.21	0.62	0.49	0.74

Coefficients >0.20 and <0.50 indicate low responsiveness, coefficients >0.5 and <0.8 indicate moderate responsiveness, and coefficients >0.8 denote high responsiveness.

Table 5 shows the means and standard deviations of the total scores of climbing up and going down stairs, considering the evaluation intervals in months and the total score at baseline.

**Table 5:** Mean and standard deviation of the total scores of climbing up and going down stairs. Evaluation intervals are represented in months (0, 3, 6, 9, and 12 months).

**Climbing up stairs**					**Going down stairs**				
**FES 0**	**FES 3**	**FES 6**	**FES 9**	**FES 12**	**FES 0**	**FES 3**	**FES 6**	**FES 9**	**FES 12**
12.82	14.17	15.82	16.43	17.34	11.84	12.65	14.15	14.69	15.23
5.09	4.65	4.57	5.32	5.85	4.76	5.78	5.90	6.49	6.99

## Discussion

This study determined the most appropriate evaluation intervals for FES-DMD-D3 (climbing up and going down stairs) by responsiveness analysis. Climbing up stairs, according to ES, ranged from low to moderate in the six-month assessments and the classification was found by SRM. Climbing down stairs showed low to moderate responsiveness in the nine-month assessments.

In addition to the FES-DMD, other scales for patients with DMD have been tested for reliability and responsiveness. One of the most commonly used is the MFM. In a study with 41 patients using SRM analysis, the total score in the MFM showed good responsiveness in the one-year interval^26^. The similarity between this study and the present study is due to the fact that both included only patients with DMD, which have significant deterioration of motor function in one year. The MFM was applied to patients with different types of spinal muscular atrophy (SMA) at three-month intervals. The authors showed that although patients had the same disease, responsiveness reflected the distinct progression of the clinical types of the disease, according to disease severity^26^.

The design and reliability testing of the FES-DMD-D3 showed that the timed performance of climbing up stairs had strong correlation with the total score. This was not true of going down stairs. The authors considered that going down stairs would require less complex movements than climbing up stairs because gravity favored descending movements^11^. In fact, climbing down stairs requires eccentric control of the extensor muscles of hips and knees, which are compromised by DMD, and as patients show reduced range of dorsal and plantar ankle flexion, the activity may become unsafe and dangerous^11^ ^,^ ^27^.

Motor strategies observed in going down stairs are more variable. Some patients used gravity to compensate decreased voluntary acceleration in the descending movement, taking a higher risk of falls. The study conducted by Martini et al.^27^ showed that the variability of compensatory movement strategies during going down stairs affects the time of execution of the activity and described two different coping strategies. Other studies reported that patients increased the number of compensatory movements and time in order to prioritize safety. In the present study, motor variability is reflected by high standard deviations.

A major limitation of the present study was the number of patients in different stages of the disease (Vignos 1-3). Patients who are closer to gait loss may have a faster clinical progression, which can affect the responsiveness of the FES-DMD-D3. Future studies should evaluate and compare the responsiveness of the FES-DMD-D3 in a larger number of patients grouped by Vignos classification. Responsiveness may vary between the groups.

## Conclusion

The FES-DMD-D3 (climbing up and going down stairs) showed responsiveness starting with the three-month assessments. Climbing up stairs showed moderate responsiveness at nine-month intervals and high responsiveness in the 12-month follow-up. Climbing down stairs showed moderate responsiveness in the 12-month follow-up.
